# Tipping the scale: Effects of physical activity and body composition on cardiac parameters in postmenopausal females

**DOI:** 10.14814/phy2.70144

**Published:** 2024-11-28

**Authors:** Mads Fischer, Andrea Tamariz‐Ellemann, Jon Egelund, Nicolai Rytter, Ylva Hellsten, Lasse Gliemann

**Affiliations:** ^1^ Department of Nutrition, Exercise and Sports University of Copenhagen Copenhagen Ø Denmark

**Keywords:** cardiac function, echocardiography, exercise, menopause, physical activity, women

## Abstract

The risk of cardiovascular disease increases significantly after menopause. We sought to assess the impact of different activity levels on cardiac structure and function in postmenopausal women. We grouped age‐similar, postmenopausal women by self‐reported physical activity levels over two decades. The study involved 34 women (age 61 ± 1 years; 11 ± 2 postmenopausal years; body mass index 23 ± 3 kg/m^2^) categorized into three activity tiers: sedentary (SED; ≤1 h exercise weekly; *n* = 9); moderately active (MOD; ≥2 ≤6 h low/moderate intensity exercise weekly; *n* = 11) and highly active (HIGH; >4 h of moderate/high intensity exercise weekly; *n* = 14). Maximum oxygen uptake (VO_2_max) differed significantly (*p* < 0.05) between the groups (24.9 ± 5.8; 30.5 ± 5.8; 38.4 ± 4.4 mL O_2_/kg/min; SED, MOD and HIGH respectively). Conversely, there were no differences (*p* > 0.05) in height, Total fat‐free mass, body surface area or in echocardiographic measures of left ventricular (LV) morphology, systolic function, diastolic function and right ventricular function. Contrary to our hypothesis, these findings reveal that marked differences in activity level and VO_2_max are not reflected in measures of LV morphology or echocardiographic indicators of cardiac diastolic or systolic function in postmenopausal women of similar body size.

## 1 INTRODUCTION

Aging is associated with a gradual decline in strength, aerobic exercise capacity, vascular and cardiac function where the effect is accelerated by lifelong physical inactivity (Celermajer et al., 1994; Fujimoto et al., 2012; Lang et al., 2015; Nyberg et al., 2014). This highlights the importance of regular exercise throughout life, in order to preserve cardiac health and maintain exercise capacity in older individuals (Arnett et al., 2019; Fujimoto et al., 2010). Women are generally better protected against age‐induced cardiovascular risks. However, this protection diminishes around midlife with menopause (Mikkola et al., 2013), which is associated with sarcopenia, occurring mainly during the first 2 years after menstruation ceases (El Khoudary et al., 2019; Janssen et al., 2002).

Few studies have evaluated the effect of aging and lifestyle on cardiac function in women, and even fewer studies have discriminated between pre‐ and postmenopausal women (Egelund et al., 2017; Nio et al., 2020). There is consequently a lack of knowledge on the extent to which cardiac function is affected by menopause and if habitual physical activity can counteract the decline in cardiovascular function that occurs in women after menopause. With advancing age, the cardiovascular system undergoes structural and functional changes that can impede cardiac health. These changes may include reduced lean body mass, decreased cardiac compliance, and ventricular hypertrophy. (Carrick‐Ranson et al., 2013; Fujimoto et al., 2012; Prasad et al., 2010). The adverse changes collectively contribute to a higher vulnerability to cardiovascular disease, particularly heart failure with preserved ejection fraction (HFpEF) which may be more pronounced in postmenopausal women (Carrick‐Ranson et al., 2013; Prasad et al., 2010). Previous studies suggest that cardiac compliance in older women improves with shorter (≤3 months) exercise training interventions (Egelund et al., 2017; Nio et al., 2020), with cross sectional studies on long‐term (≥1 year) exercise training showing even greater effects (Carrick‐Ranson et al., 2020; Fujimoto et al., 2010; Howden et al., 2018).

In addition, a clear association persist between maximum oxygen uptake (VO_2_max) and cardiac size and function both in absolute terms and when correcting for body surface area (BSA) (Carrick‐Ranson et al., 2013; Carrick‐Ranson et al., 2020; Foulkes et al., 2023; Zong et al., 2014). However, if assessing the fat‐free body mass (FFM), postmenopausal women show nearly identical measures of blood volume, left ventricular (LV) mass, and LV end‐diastolic volume (EDV) (Carrick‐Ranson et al., 2020), despite markedly different VO_2_max. Given the strong associations between cardiac morphology and FFM (Carrick‐Ranson et al., 2020; Gomes et al., 2023), studies utilizing the standard indexing methods (e.g., weight or BSA) may systematically overstate the differences in cardiac morphological adaptations in athlete populations while understating the same measures in sedentary and obese. This consideration is particularly important given the negative correlation between adipose mass and these cardiac metrics (Carrick‐Ranson et al., 2013). Such biases may explain the findings of studies like that of Foulkes et al. (2023) showing that LV EDV was a strong predictor of VO_2_max whereas BSA indexing of LV EDV reduced the strength of the prediction by over adjusting for low‐metabolic tissue (i.e., fat mass) (Carrick‐Ranson et al., 2013; Gomes et al., 2023; Letnes et al., 2023).

The aim of this study was to assess the effect of habitual physical activity levels on echocardiographic measures of cardiac function in postmenopausal females and to evaluate the relationship between cardiac structure, function, and VO_2_max. The hypothesis was that cardiac structural and functional differences would be related to lifelong physical activity levels and VO_2_max.

## 2 METHODS

### 2.1 Ethical approval

The study was part of a larger project on cardiovascular function in postmenopausal women, approved by the Ethics Committee of Copenhagen and Frederiksberg communities (H‐16042441). It was conducted in accordance with the *Declaration of Helsinki*. Written informed consent was obtained before enrollment. The subjects in this study are a subgroup of a cohort previously characterized for studies on peripheral vascular function (Gliemann et al., 2020, 2021).

### 2.2 Subjects

Thirty‐four healthy postmenopausal women participated in the current study (age of 61 ± 4 years; BMI of 23 ± 3; postmenopausal 11 ± 5 years, Table 1). Menopausal status and methods for assessing health status have been described previously (Gliemann et al., 2020, 2021). Postmenopausal women with normal ECG results were recruited for the study. All participants had to be capable of providing informed consent. The exclusion criteria were as follows: having had a menstrual cycle within the past year of the date of participation, a BMI over 29.9, systolic blood pressure of 140 mmHg or higher, or diastolic blood pressure of 90 mmHg or higher. Individuals with chronic diseases, cardiovascular conditions, cancer within the last 5 years, liver or kidney disease, or a smoking history in the past 10 years were excluded. Participants were also ineligible if they consumed more than seven units of alcohol per week or had ever undergone hormone replacement therapy (except for local treatments). Additionally, those currently involved in other clinical trials were not eligible. All participants were unmedicated during the study.

**TABLE 1:** Subject characteristics.

								Tukey's multiple comparisons test		
Group	Unit	SED (*n* = 9)		MOD (*n* = 11)		HIGH (*n* = 14)		SED vs. MOD	MOD vs. HIGH	SED vs. HIGH
Age	Years	61.8 ± 4.1		61.4 ± 4.2		60.1 ± 4.3		0.974	0.757	0.644
Time post menopause	Years	11 ± 4		12 ± 6		10 ± 5		0.952	0.481	0.709
Height	cm	170 ± 4		166 ± 5		168 ± 6		0.251	0.453	0.842
Body Mass	kg	69.7 ± 12.8		66.6 ± 6.5		60.3 ± 4.9		0.688	0.147	**0.030**
Fat percentage	%	37.8 ± 6.2		37.3 ± 6.9		29.6 ± 4.6		0.983	**0.007**	**0.007**
Fat‐free body mass	kg	42.7 ± 4.9		42.0 ± 4.2		42.6 ± 4.8		0.817	0.994	0.837
Body surface area	m^2^	1.79 ± 0.17		1.74 ± 0.09		1.68 ± 0.09		0.516	0.489	0.078
Resting heart rate	Beats/min	67.6 ± 6.7		62.6 ± 8.5		58.3 ± 11.8		0.493	0.079	0.520
VO_2max_	(mL/min)	1730 ± 478		2101 ± 322		2319 ± 332		0.107	0.375	**0.003**
VO_2_ per kg	(mL/min/kg)	25.1 ± 5.2		31.9 ± 5.9		38.4 ± 4.4		**0.017**	**0.045**	**<0.001**
VO_2_ per FFM	(mL/min/kg)	40.3 ± 6.5		50.1 ± 5.3		54.5 ± 6.6		**0.007**	0.331	**<0.001**
Maximal heart rate	Beats/min	165 ± 13		173 ± 14		169 ± 11		0.371	0.776	0.689
Systolic blood pressure	mmHg	140 ± 24		129 ± 18		121 ± 16		0.379	0.550	0.056
Diastolic blood pressure	mmHg	80 ± 8		79 ± 8		75 ± 9		0.969	0.429	0.333
Classification of blood pressure ESH/ESC (Williams et al., 2017)	Grade	Normal	4	Normal	8	Normal	12	0.055	0.656	**0.006**
		Grade 1	2	Grade 1	3	Grade 1	2			
		Grade 2	3	Grade 2	0	Grade 2	0			

*Note*: Subject characteristics: Characteristics of healthy sedentary (SED), moderately active (MOD) and highly active (HIGH) postmenopausal female subjects. V̇O_2_max, Maximal pulmonary oxygen consumption; FFM, fat‐free body mass; Significant values highlighted in bold.

### 2.3 Study design

A cross‐sectional design was used, stratifying subjects into three groups based on their current activity levels: sedentary (SED) (<1 h weekly light exercise and no moderate or strenuous exercise), moderately active (MOD) (2–4 h of light/moderate exercise or 1–2 h of strenuous exercise weekly), and highly active (HIGH) (>4 h of moderate/strenuous exercise weekly). The number of subjects in each group was determined a priori using power and effect size calculations from our previous study by Gliemann et al. (2020). The sample size was considered sufficient, as power calculations indicated that a total of 10 subjects is required to achieve adequate power (0.95) for E/A ratio assessments (Egelund et al., 2017).

### 2.4 Anthropometrics and cardiorespiratory fitness

Body composition was assessed by full‐body dual‐energy *x*‐ray absorptiometry scanning (DXA; Prodigy, GE Healthcare, Chalfont St. Giles, UK). Following this procedure, subjects' cardiac function and morphology were assessed as explained in detail below.

VO_2_max was assessed using the Oxycon Pro (Viasys Healhtcare, Hoechberg, Germany) during an incremental exercise on a cycle ergometer (Monark Ergomedic 839E; Monark, Vansbro, Sweden). The incremental test consisted of 8 min of warm‐up session at 50, 70, or 90 W (SED, MOD and HIGH, respectively) after which workload was increased from their warm‐up intensity by 20 W per minute until exhaustion. Subjects received verbal instructions and encouragements throughout the bicycle exercise test. VO_2max_ was calculated from the highest consecutive 15 s intensity period as qualified from the three following criteria: Individuals inability to maintain a cadence of >80 revolutions per minutes; a heart rate close to predicted maximum that is, 220 minus age (Fox & Haskell, 1970); and plateau of the VO_2_ curve with a respiratory exchange rate >1.15. Data from these measurements have previously been reported (Gliemann et al., 2020) and are solely used here for the purpose of relation to cardiac measurements.

### 2.5 Echocardiographic assessment

Transthoracic echocardiography was performed using a GE Vivid E9 ultrasound machine with a 2.5‐MHz transducer (GE Healthcare). Subjects were examined with the same protocol as previously described (Egelund et al., 2017) according to current echocardiographic guidelines by the ESCI and the ASE (Lang et al., 2015). LV mass was calculated using the cube formula, while volumetric measurements were evaluated using Simpsons Biplane method. The measurements were analyzed on a computer using EchoPac (V113 GE Healthcare) blinded and in unidentifiable order by one investigator (J.E).

### 2.6 Measurements and calculations

Indexed measures are calculated as main variable divided by either body BSA (Dubois and Dubois formula) or FFM in order to normalize for body size (Carrick‐Ranson et al., 2013; Gomes et al., 2023; Lang et al., 2015; Zong et al., 2014).

A linear mixed‐model approach was used to investigate the effect of different activity levels in the three groups. Subjects were specified as a repeated factor and identifier of random variation. Post‐hoc procedure was used to detect pairwise differences, performed with multiple comparison, and single‐step adjusted *p*‐values are reported. Statistical analyses were performed with R (version 4.1.2; R, Vienna, Austria) using the interface RStudio (build 554; RStudio Team, Boston, USA). Power and effect size calculations were performed using G*Power (Faul et al., 2007). Data are reported as mean ± standard deviation (SD). Alpha was set at the 0.05 level.

## 3 RESULTS

The three groups were of similar age, height and menopausal time, but reported habitual physical activity levels were different between all groups (*p* ≤ 0.005) and body mass was different (*p* = 0.03) between SED and HIGH (Table 1). The difference in body mass between SED and HIGH were caused by differences in total body fat mass with FFM being similar between all groups (Table 1). VO_2_max were significantly different between the three groups and the difference was still evident after correcting for total body mass and FFM (Table 1).

**FIGURE 1:**
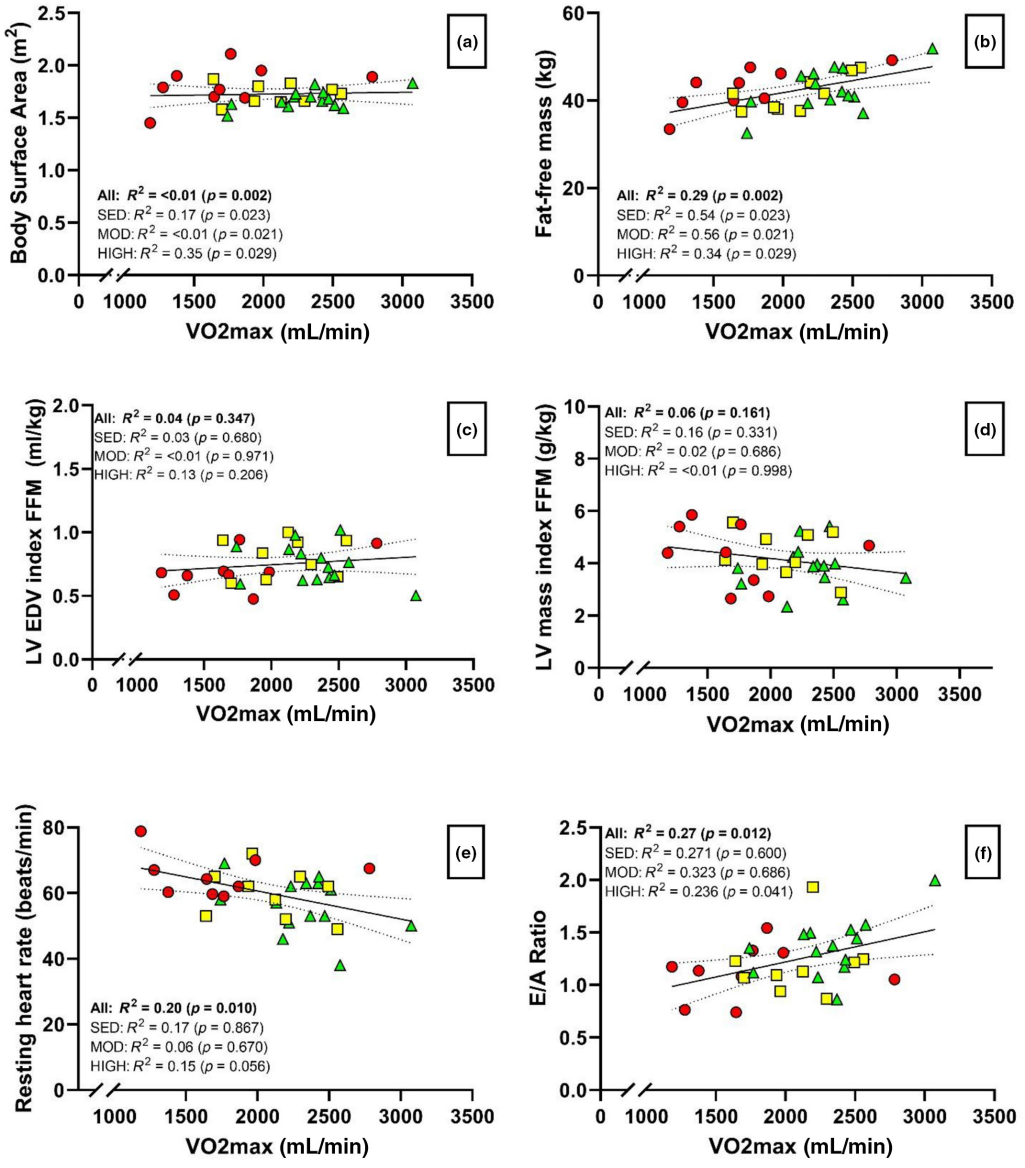
Linear regression for association between key echocardiographic parameters indexed to fat‐free body mass (FFM) and/or VO_2_max. (a) Body surface area per VO_2_max; (b) VO_2_max per FFM; (c) left ventricle (LV) end‐diastolic volume (EDV) per VO_2_max; (d) LV mass indexed to FFM per VO_2_max; (e) Resting heart rate (BPM) per VO_2_; (f) Mitral valve E/A ratio per VO_2_max. Open circles (○) show sedentary (SED). Squares (■) show moderately active (MOD) and triangles (▲) show Highly active (HIGH). Regression lines show the trend of all data (i.e., combined group trends) with their 95% confidence interval.

*Cardiac morphology and volumes* No group differences were found in measures of absolute or indexed LV mass and dimensions (Table 2). LV end‐diastolic volume, end‐systolic volume and LV mass were similar between all three groups as well (Table 2) with no correlation between LV end‐diastolic volume, LV mass and VO_2max_ relative to FFM (Figure 1). *Cardiac systolic function*: The three groups had similar global longitudinal strain as well as ejection fraction and MV s' (Table 2). *Cardiac diastolic function*: Mitral E/A ratio showed a tendency to be greater in the HIGH group compared to SED and MOD but were not significantly difference between groups (Table 3). However, there was a small positive correlation (*R* ^2^ = 0.27, *p* = 0.024) between E/A ratio and FFM adjusted VO_2max_ (Figure 1). Likewise, resting heart rate showed a tendency to be lower in the HIGH group compared to SED and MOD but were not significantly difference between groups (Figure 1) while a small negative correlation (*R* ^2^ = 0.20, *p* = 0.010) between resting heart rate and VO_2max_ (Figure 1). *Left atrium morphology*: No differences were found in absolute or relative left atrial size (Table 2). *Right ventricular function*: No differences between any of the measured markers of right ventricular function (TAPSE, S′) were found.

**TABLE 2:** Morphology and dimensions.

Group	Unit	SED (*n* = 9)	MOD (*n* = 11)	HIGH (*n* = 14)	Tukey's multiple comparisons test		
					SED vs MOD	MOD vs HIGH	SED vs HIGH
Left ventricle							
Internal dimension	cm	4.61 ± 0.4	4.46 ± 0.3	4.40 ± 0.5	0.432	0.737	0.256
Septal wall thickness	cm	1.03 ± 0.1	1.02 ± 0.2	0.98 ± 0.1	0.742	0.616	0.414
Posterior wall thickness	cm	0.76 ± 0.2	0.79 ± 0.1	0.81 ± 0.1	0.672	0.720	0.435
Relative wall thickness	Equation 2	0.33 ± 0.07	0.36 ± 0.06	0.38 ± 0.10	0.786	0.788	0.395
Mass	gram	138 ± 21	134 ± 34	128 ± 27	0.742	0.616	0.414
Mass index BSA	g/m^2^	77 ± 14	76 ± 18	76 ± 14	0.903	0.945	0.847
Mass index FFM	g/kg	4.3 ± 1.2	4.6 ± 0.9	3.9 ± 0.9	0.852	0.175	0.484
End‐diastolic volume	mL	96 ± 27	100 ± 15	94 ± 16	0.632	0.424	0.801
End‐systolic volume	mL	38 ± 11	43 ± 7	39 ± 8	0.246	0.196	0.996
Stroke Volume	mL	58 ± 19	58 ± 9	56 ± 12	0.968	0.732	0.715
Stroke Volume index BSA	mL/m^2^	32 ± 3	33 ± 2	33 ± 2	0.960	0.997	0.934
Stroke Volume index FFM	mL/kg FFM	1.4 ± 0.4	1.4 ± 0.2	1.3 ± 0.3	0.965	0.804	0.942
Cardiac Output	L/min	3.9 ± 1.2	3.6 ± 0.7	3.3 ± 1.1	0.819	0.676	0.336
Cardiac Output index BSA	L/min/m^2^	2.2 ± 0.6	2.1 ± 0.4	1.9 ± 0.6	0.919	0.882	0.667
Cardiac Output index FFM	(mL/min/kg FFM)	96.5 ± 24	86.9 ± 18	77.5 ± 25.3	0.643	0.565	0.162
Left atria size							
LAEDV	mL	56.3 ± 24	65.8 ± 13	65.7 ± 16	0.240	0.982	0.224
LAEDV index BSA	mL/m^2^	31.1 ± 12	37.7 ± 8	39.1 ± 9	0.138	0.736	0.064
LAEDV index FFM	mL/kg FFM	1.3 ± 0.5	1.6 ± 0.3	1.6 ± 0.4	0.260	0.975	0.314
LAESV	mL	28.2 ± 13	33.0 ± 6	30.8 ± 7.6	0.234	0.539	0.495
LAESV index BSA	mL/m^2^	15.6 ± 7	19.0 ± 4	18.4 ± 4.5	0.140	0.772	0.197
LAESV index FFM	mL/kg FFM	0.7 ± 0.3	0.8 ± 0.2	0.7 ± 0.2	0.297	0.707	0.679

*Note*: Values are mean ± SD.

Abbreviations: EF indicates ejection fraction; FFM, fat‐free body mass; IVSd, left ventricular interventricular septum diastole; LV, left ventricular; LVEDV, left ventricular end‐diastolic volume; LVESV, left ventricular end‐systolic volume; LVIDd, left ventricular end‐diastolic diameter; LVPWd, left ventricular posterior wall thickness diastole.

**TABLE 3:** Echocardiographic dynamic variables.

Group	Unit	SED (*n* = 9)	MOD (*n* = 11)	HIGH (*n* = 14)	Tukey's multiple comparisons test		
					SED vs MOD	MOD vs HIGH	SED vs HIGH
Systolic function							
Ejection fraction	%	60.0 ± 6	57.5 ± 3	59.3 ± 6	0.306	0.421	0.747
Global Longitudinal Strain	%	−19.2 ± 3.3	−19.6 ± 2.1	−19.8 ± 1.7	0.906	0.979	0.805
Pulsed TDI s'	cm/s	8 ± 1.43	7.23 ± 1.02	7.7 ± 0.63	0.121	0.332	0.458
Diastolic function							
Mitral valve E velocity	m/s	0.72 ± 0.07	0.84 ± 0.11	0.76 ± 0.08	**0.007**	**0.042**	0.316
Mitral valve A velocity	m/s	0.67 ± 0.2	0.75 ± 0.1	0.57 ± 0.1	0.166	**0.001**	0.094
Mitral inflow velocity E/A	ratio	1.13 ± 0.3	1.15 ± 0.3	1.36 ± 0.3	0.875	0.063	0.056
Mitral deceleration time	ms	196 ± 46	207 ± 36	196 ± 52	0.584	0.541	1.0
Pulsed TDI e'	cm/s	7.2 ± 1.5	7.4 ± 1	8.3 ± 1	0.951	0.183	0.268
Isovolumic relaxation time	ms	95 ± 13	99 ± 14	96 ± 9	0.508	0.535	0.910
Right ventricle function							
TAPSE	cm	2.54 ± 0.2	2.66 ± 0.4	2.57 ± 0.3	0.428	0.530	0.807
Pulsed TDI s'	cm/s	13.0 ± 1.6	13.3 ± 1.6	13.7 ± 1.52	0.694	0.558	0.336

*Note*: Echocardiographic characteristics of healthy sedentary (SED), moderately active (MOD) and highly active (HIGH) female subjects. Values are mean ± SD.

Abbreviations: PW TDI, pulsed wave tissue doppler imaging; TAPSE, tricuspid annular plane systolic excursion.

## 4 DISCUSSION

The primary aim of the current study was to determine the impact of life‐long physical activity on cardiac function in postmenopausal women. Contrary to our hypothesis, the three groups of women who were stratified by physical activity levels, presented remarkably similar echocardiographic measures both in terms of function and morphology. Despite this lack of difference at the cardiac level the well‐trained women presented a 34% higher VO_2_max than the sedentary group. Assessment of the relations of VO_2_max and echocardiographic measures of ventricular compliance (i.e., E/A ratio) found significant correlations herein, whereas group comparisons did not reveal any differences. The recruited subjects were remarkably similar in terms of anthropometric measurements such as height, BSA and FFM, while differing significantly in fat percentage and VO_2_max.

A main determinant of endurance exercise performance is cardiac output, the product of heart rate and stroke volume (Carrick‐Ranson et al., 2014). Since maximal heart rate is not trainable, factors that determine stroke volume are of greatest importance for exercise performance. Training adaptations of the heart are not only morphological, but also functional as evidenced when cardiac function is assessed during examinations with exercise stress, pharmacological stress or during manipulations with filling pressures (Fischer et al., 2022; Foulkes et al., 2023; Levine et al., 1991). In this study, we observed that although the VO_2_max of the highly trained group significantly exceeded that of the moderately trained and sedentary groups, differences in cardiac morphology among the groups were diminished by indexing to FFM. To our knowledge, this is the first study to demonstrate that among age‐matched postmenopausal women, morphological, and resting functional characteristics across varying physical activity levels are markedly diminished when controlling for essential anthropometric variables, such as FFM. This finding is supported by data from Carrick‐Ranson et al. (2020) showing that differences in LV EDV, stroke volume, and LV mass (Table 2 and Figure 1) are nulled when indexing to FFM.

Cardiac function declines with age, but the decline has mainly been assessed in large populations which often include both males and females (Fujimoto et al., 2012; Scalia et al., 2010). Studies assessing the effect of age alone on resting cardiac function using echocardiography in women show inconsistent or even paradoxical data as illustrated in a study by Scalia et al., where the within age‐group 5 year decline in ejection fraction was greater than the between age group differences (Scalia et al., 2010). Adding to this, differences in echocardiographic measures of cardiac systolic function can be difficult to detect as they can be masked by the differences in resting heart rate and/or arterial pressure between subjects (MacNamara et al., 2021; Quintana et al., 2005). The same problem may apply to assessment of cardiac diastolic function, as evidenced by studies showing diverging results in the correlation between invasive measures of intracardiac filling pressure and echocardiographic markers hereof (i.e., MV E/A and E/e´ ratio) (Pluim et al., 2000; Prasad et al., 2007). While several studies show improved diastolic function with long term exercise training (Fujimoto et al., 2012; Howden et al., 2018; Levine et al., 1991), we found a weak, yet significant correlation between VO_2_max and E/A ratio but none with left ventricle morphology, or any other diastolic and systolic function parameters (Table 3). Thus, standard echocardiographic measures of cardiac function collected at rest are not well suited to differentiate between healthy individuals with varying fitness levels (Bhella et al., 2014; Fischer et al., 2022). Instead, volume loading and or exercise stress‐testing to challenge cardiac function is more likely to reveal functional differences as has been shown by others (Carrick‐Ranson et al., 2020; Foulkes et al., 2023; Nio et al., 2020; Prasad et al., 2010).

Scaling of biological systems are crucial when investigating and evaluating between normal, super and pathophysiology, as biological systems are naturally scaled to total body mass (Zong et al., 2014). The unexpected lack of difference in cardiac dimensions between the three groups of women warrant some further considerations. While utilizing body mass for normalization of VO_2max_, most studies in cardiac morphology have utilized BSA for scaling (Bhella et al., 2014; Dogra et al., 2012). However, well‐trained populations have a lower fat mass and a greater FFM compared to a sedentary individual. In the present study, body mass and fat percentage was lower in the well‐trained compared to the sedentary group, which could in part have influenced the results, although FFM was similar. These differences in body composition makes BSA a less optimal ‘normalization’ index when comparing trained versus untrained as total muscle mass has more pronounced associations for cardiac morphology and function than both BSA, fat mass, height and total body mass (Bella et al., 1998; Carrick‐Ranson et al., 2013; Gomes et al., 2023). Previous studies of similar age and gender have also found less variation in key cardiac parameters, when correcting for FFM, but with paradoxically smaller ventricular size when normalized for BSA (Carrick‐Ranson et al., 2013; Gomes et al., 2023). While noting the feasibility of BSA which is more commonly used in clinical practice, this is a sub‐optimal tool for normalization of cardiovascular parameters considering the lack of physiological relevance of total android fat mass on cardiac dimensions (Carrick‐Ranson et al., 2013; Gomes et al., 2023).

The large difference in VO_2_max between the three groups of women and a similar maximal heart rate suggests that there must have been a 24% difference in maximal stroke volume between the sedentary and the well‐trained groups (Proctor et al., 1985). This estimate is supported by a large body of literature showing that the relationship between cardiac output and pulmonary O_2_ uptake are linear (Carrick‐Ranson et al., 2014; Proctor et al., 1985) and that systemic oxygen utilization are not altered by fitness or age (Ogawa et al., 1992; Stratton et al., 1994). However, the echocardiographic data on resting cardiac function shown in the current study (Table 3) do not reflect the difference in maximal stroke volume and cardiac output required to explain the observed differences in VO_2_max. These findings indicate that the key distinctions in cardiac function among highly active, moderately active, and sedentary postmenopausal women are related to adaptations in ventricular compliance which become apparent primarily during altered filling‐pressures and exercise stress rather than at during rest (Egelund et al., 2017; Nio et al., 2020; Levine et al., 1991). This highlights the critical need for assessing cardiac function under exercise conditions to capture dynamic changes not detectable during resting measurements.

## 5 LIMITATIONS

Given that echocardiographic measures are inherently vulnerable to subjectivity, relying on a single observer introduces the possibility of systemic bias. While all measurements were performed blinded and with care and following standardized protocols, the lack of inter‐observer comparison reduces the ability to assess variability in measurement.

The E/A ratio was chosen for the power analysis due to its well‐established role in assessing diastolic function, particularly in the context of exercise‐induced adaptations (Bhella et al., 2014; Weiner et al., 2015). This decision was primarily methodological, aiming for consistency with our previous research (Egelund et al., 2017; Schmidt et al., 2013, 2014), and because the E/A ratio provides a clear threshold for identifying early signs of diastolic dysfunction. Given the specific design of this study, which aimed to assess group‐level differences in diastolic function, the E/A ratio served as an appropriate marker based on its sensitivity to changes induced by exercise interventions. However, we acknowledge that the E/A ratio is not without its limitations. While E/A show good continuous association with BMI and VO_2_max (Brinker et al., 2014; von der Born et al., 2022) it may not alone fully capture the spectrum of diastolic dysfunction (Nagueh et al., 2016), particularly in distinguishing between its intermediate stages.

In light of these limitations, while the study's findings provide valuable insights into the relationship between exercise and diastolic function, we encourage further research using larger sample sizes to validate and extend our findings.

## 6 CONCLUSIONS

In conclusion, this study demonstrates that lifelong physically active postmenopausal women exhibit similar cardiac morphology and function to their sedentary counterparts. These findings underscore the need to assess cardiac function under stress conditions, preferably through exercise stress testing. Additionally, more research on cardiac function should include measures of FFM, as current standards indexed by whole body weight may introduce systematic bias when comparing well‐trained to sedentary individuals.

## AUTHOR CONTRIBUTIONS

Gliemann and Hellsten conceptualized the study. Data collection was performed by Gliemann, Egelund, Tamariz‐Ellemann and Rytter. Data analysis and interpretation was performed by Fischer, Gliemann, Tamariz‐Ellemann and Hellsten, and Fischer and Gliemann drafted the manuscript. All authors critically revised the manuscript and approved its last version.

## FUNDING INFORMATION

No funding information provided.

## CONFLICT OF INTEREST STATEMENT

The authors have no conflicts of interest to disclose. The results of the study are presented clearly, honestly, and without fabrication, falsification, or inappropriate data manipulation.

## Data Availability

The data published in this study are available upon reasonable request and must be in accordance with current General Data Protection Regulation (GDPR).
